# Does repeat Hb measurement within 2 hours after a normal initial Hb in stable trauma patients add value to trauma evaluation?

**DOI:** 10.1186/s12245-014-0026-3

**Published:** 2014-07-10

**Authors:** Joanne C Sierink, Pieter Joosse, Steve MM de Castro, Niels WL Schep, J Carel Goslings

**Affiliations:** 1 Trauma Unit, Department of Surgery, Academic Medical Center, Meibergdreef 9, Amsterdam AZ 1105, The Netherlands

**Keywords:** Trauma, Injury, ABG, Routine, Laboratory, Haemoglobin

## Abstract

**Background:**

In our level I trauma center, it is considered common practice to repeat blood haemoglobin measurements in patients within 2 h after admission. However, the rationale behind this procedure is elusive and can be considered labour-intensive, especially in patients in whom haemorrhaging is not to be expected. The aim of this study was to assess the value of the repeated Hb measurement (r-Hb) within 2 h in adult trauma patients without evidence of haemodynamic instability.

**Methods:**

The local trauma registry was used to identify all trauma patients without evidence of haemodynamic instability from January 2009 to December 2010. Patients in whom no initial blood Hb measurement (i-Hb) was done on admission, referrals, and patients without risk for traumatic injuries or haemorrhage based upon mechanism of injury (e.g. inhalation or drowning injury) were excluded.

**Results:**

A total of 1,537 patients were included in the study, 1,246 of which did not present with signs of haemodynamic instability. Median Injury Severity Score (ISS) was 5 (interquartile range (IQR) 1 to 13), 22% of the patients were multitrauma patients (ISS > 15). A normal i-Hb was found in 914 patients (73%). Of the 914 patients with a normal i-Hb, 639 (70%) had a normal r-Hb, while in 127 patients (14%), an abnormal r-Hb was found. In none of these patients, the abnormal r-Hb led to new diagnoses. In 148 patients (16%), no repeated Hb measurement was done without clinical consequences.

**Conclusion:**

We conclude that repeated blood Hb measurement within 2 h after admission in stable, adult trauma patients with a normal initial Hb concentration does not add value to a trauma patient's evaluation.

## Background

The Advanced Trauma Life Support (ATLS) guidelines provide a structured approach to examine trauma patients according to the principle ‘treat first what kills first’ [1]. A blood haemoglobin (Hb) measurement is part of this structured approach and is obtained during the primary survey. Neither in the ATLS guidelines, nor in the EAST guidelines is repeating haemoglobin measurement mentioned, although ‘continuous monitoring of vital signs and urinary output by using arterial blood gas analyses and cardiac monitoring devices’ is advised as part of the secondary survey.

In our level I trauma centre, it is considered common practice to repeat blood haemoglobin (Hb) concentration measurements in patients within 2 h after admission. Nor in the ATLS guidelines, nor in EAST guidelines it is stated that a repeated Hb blood measurement should be done. This habit of repeated Hb measurement is not widespread in hospitals in North America. However, it is done in numerous hospitals in The Netherlands in order to detect occult bleeding in patients without evidence of haemodynamic instability. While the association between mortality and haematological laboratory parameters in (trauma) patients visiting the emergency department has been described in previous studies [2]-[4], these studies have not reported Hb to have an association with mortality in trauma patients. It is an interesting finding in itself that this local habit is done without solid scientific evidence.

The aim of this study was to assess the value of a repeated blood Hb measurement within 2 h in adult trauma patients without evidence of haemodynamic instability.

## Methods

We investigated the clinical consequences of a repeated blood Hb measurement within 2 h after hospital admission in a cohort of adult haemodynamic stable trauma patients.

### Patients and data collection

A prospective, comprehensive registration is maintained of all trauma patients admitted at our Level I trauma centre. From this trauma registry, all patients treated at the trauma (resuscitation) room from January 2009 to December 2010 were identified. Patients under 16 years of age were excluded. Evidence of haemodynamic instability on arrival was also considered an exclusion criterion. Haemodynamic instability was defined as a systolic blood pressure below 90 mmHg [5],[6] and/or a heart rate higher than 120 beats per minute on admission. If the heart rate only was abnormal or if vital signs were missing from the database, a state of haemodynamic instability was ascribed to the patient if the attending trauma surgeon had cited the following terms (or synonyms thereof) in the medical chart: hemodynamic instability, evidence for haemodynamic instability or rapid clinical deterioration.

Excluded were patients in whom no initial blood Hb measurement was done on admission, referrals, patients without risk for traumatic injuries or haemorrhage based upon mechanism of injury (inhalation, drowning or dive injury, intoxications, electrocution and suicide attempt by hanging) and patients with an abnormal Hb on admission.

The collected data included age, gender, mechanism of injury, Injury Severity Score (ISS), duration of hospital and ICU-stay and in-hospital mortality. Laboratory data consisted of initial Hb and repeat Hb levels in grams per deciliter (g/dl). Patients with a normal initial Hb were divided into three groups: patients with a repeat Hb level within normal range, patients with an abnormal repeat Hb level and patients in whom no repeat Hb measurement was performed.

### Definitions

The I-Stat (Abbot, Abbot Park, IL, USA) or Sysmex XE5000 (Sysmex, the Netherlands) was used for all Hb measurements as is the routine at our institution. Reference values for blood Hb concentration in our centre are 12.1 to 16.1 g/dl for women and 13.7 to 16.9 g/dl for men. Initial blood Hb concentration was defined as the first Hb measurement done in the trauma room and was further described as initial Hb (i-Hb). Abnormal i-Hb concentration was defined as a value below the minimum of the reference range. Repeated blood Hb concentration was defined as a sample obtained within 2 h following initial Hb measurement and was described as repeated Hb (r-Hb) level. An abnormal r-Hb level was defined as a value outside the reference range or a significant decrease in Hb level. A significant decrease was calculated with the following formula [7]:

(1) $$\mathrm{Critical}\;\mathrm{difference}\;\left(\%\right)=2.77\;x\surd \left({\left(\mathrm{CVa}\right)}^{2}+{\left(\mathrm{CVb}\right)}^{2}\right)$$

This equation was chosen because it combines the analytic variation coefficient (CVa; 1.2% for both blood analyzers) and the biologic variation coefficient of haemoglobin (CVb; 2.8%) [8]. This results in a critical difference of 8.4%. The i-Hb and r-Hb therefore differ significantly if the decrease is at least 8.4% of the i-Hb. This will be referred to as a ‘significant decrease’ in Hb concentration.

An abnormal r-Hb suggesting either unidentified injuries or deterioration of existing injuries was considered of clinical consequence. Whether a decrease in Hb directly lead to surgical therapy, radiological intervention or the necessity for a blood transfusion could not be assessed based solely on the r-Hb. However, patients who underwent an emergent surgical or radiological procedure to control haemorrhage were identified and analysed.

Imaging in our study population had been performed according to ATLS guidelines and consisted of chest and pelvic X-rays, Focussed Assessment with Sonography in Trauma (FAST) followed by selective CT scanning [1]. Between January 2009 and April 2011, patients in whom severe injury was suspected based on predefined vital signs and clinically suspicious diagnoses, underwent immediate total-body CT scanning as a pilot study for the REACT-2 trial [9],[10].

### Statistics

Descriptive statistics with SPSS software (version 18.0; SPSS Inc, Chicago, IL, USA) were used to describe the data. Data were expressed as number (%) or median (interquartile range (IQR)), or mean (standard deviation) in case of normally distributed data. Categorical data were compared using *χ*^2^ analyses. A *p* value < 0.05 was considered significant.

## Results

During the study period, 1,537 patients were evaluated at the trauma (resuscitation) room. Patients under 16 years of age were excluded (*n* = 143). There were 57 patients with evidence of haemodynamic instability on arrival: 29 patients had a lowered systolic blood pressure. Fourteen patients had an increased heart rate and evidence for haemodynamic instability was described in the medical charts. In 14 patients, data on blood pressure or pulse were not registered. These patients sustained the following conditions: cardiopulmonary resuscitation on admission due to traumatic injuries (*n* = 10), immediate operative treatment due to a major intraventricular bleed or acute subdural hematoma (*n* = 2), traumatic placental abruption in an assaulted pregnant patient (*n* = 1) and immediate clinical deterioration and death due to severe traumatic brain injury (*n* = 1).

Referrals (*n* = 39) and patients that had sustained specific injury mechanisms (*n* = 31) were excluded as well. In 21 patients, no Hb measurement was done on admission (due to a low-energy trauma, *n* = 3; a high-energy trauma after which the patient was discharged within 12 h, *n* = 12; because the patient was non-cooperative, *n* = 3; and for unknown reasons, *n* = 3).

**Figure 1:**
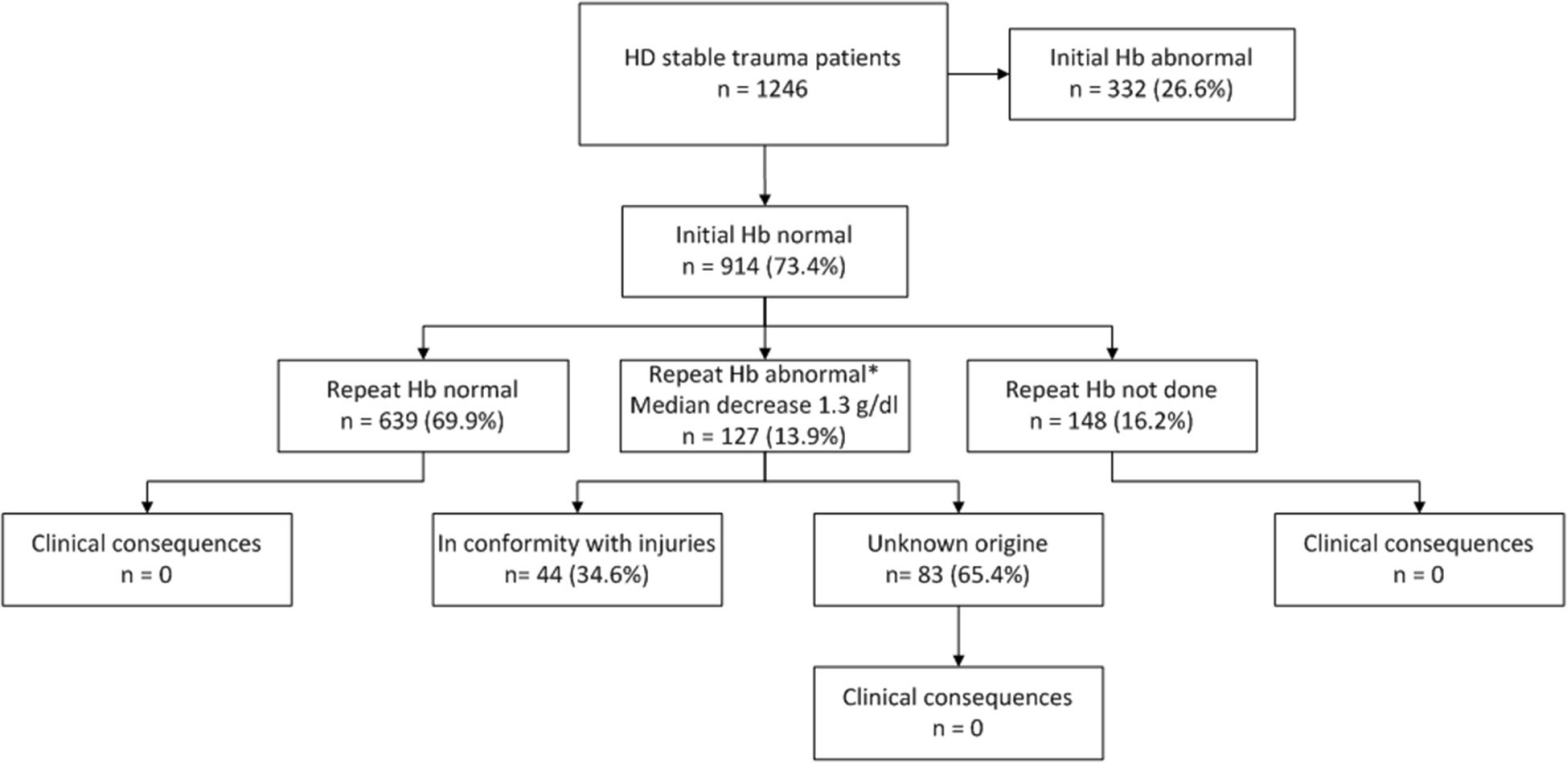
Flowchart of repeat Hb measurement within 2 h in trauma patients without evidence of haemodynamic instability.

A flowchart of the r-Hb measurements in the remaining 1,246 trauma patients is shown in Figure 1. Hundred twenty nine of the patients underwent total-body CT scanning (10%). Nine hundred and fourteen (74%) patients had a normal i-Hb level. Repeated blood Hb measurement in patients with a normal i-Hb was performed in 766 patients (84%). In 582 patients (76%), this measurement was performed within the hour, and in 184 (24%) patients, it was performed within 1 to 2 h after admission to the trauma room. There was no significant difference in abnormal r-Hb measurements for patients whose Hb was measured within 2 h and between 1 to 2 h (*P* = 0.140).

Table 1 depicts the patient demographics of the total group of 1,246 patients and the analysed group of 914 patients with a normal i-Hb.

**Table 1:** Patient demographics

	**HD stable**	**HD stable + initial Hb normal**
	***n*** **= 1,246**	***n*** **= 914**
Age (years)	40.6 (18.0)*	39.4 (16.4)*
Men	812 (65.3%)	578 (63.2%)
Blunt trauma	1,160 (93.3%)	862 (94.3%)
ISS (points)	5 (1 to 13)	5 (1 to 10)
AIS code severity 3 or more/total AIS codes	907/2,994 (30.3%)	495/1,878 (26.4%)
AIS head ≥ severity 3	351 (39%)	206 (41.8%)
AIS chest ≥ severity 3	142 (15.8%)	75 (15.2%)
AIS abdomen ≥ severity 3	120 (13.3%)	74 (15%)
AIS extremities ≥ severity 3	222 (24.5%)	104 (21%)
Multitrauma (ISS ≥ 16)	270 (21.7%)	149 (16.3%)
GCS on admission	15 (14 to 15)	15 (15 to 15)
Initial Hb (g/dl)		
Men	14.2 (1.5)*	15.0 (0.9)*
Women	12.8 (1.5)*	13.4 (0.9)
Repeat Hb in g/dl		
Men	13.9 (1.7)*	14.6 (1.1)*
Women	12.5 (1.6)*	13.1 (1.0)*
Hospital stay (days)	2 (0 to 5)	2 (0 to 4)
ICU stay (days)	0 (0 to 0)	0 (0 to 0)
In-hospital mortality	32 (2.6%)	12 (1.3%)

Data are number (%) or median (interquartile range (IQR)) unless otherwise indicated. *Mean (SD). HD, hemodynamically; Hb, haemoglobin; ISS, Injury Severity Score; AIS, Abbreviated Injury Scale; GCS, Glasgow Coma Score; g/dl, grams per deciliter; ICU, Intensive Care Unit.

Of the patients with a normal i-Hb there were 639 patients with a normal r-Hb (70%), 127 patients with an abnormal r-Hb (14%), and 148 patients in whom no r-Hb was done (16%) (Figure 1).

In none of the patients with a normal r-Hb, there were unsuspected events reported.

The abnormal r-Hb did not have clinical consequences for any of the 127 patients. In 44 patients (35%), the abnormal r-Hb corresponded with injuries found on radiologic imaging. Those patients had a median ISS of 21 (IQR = 11 to 29) with a median total hospital stay of 9 (IQR = 4 to 16). In 83 patients (65%), the cause of the abnormal r-Hb remained unclear; in 34 patients, a third Hb measurement was not performed; in 27 patients, the third Hb was normal and in 22 patients, the third Hb was abnormal without an indication for severe traumatic injuries.

Unsuspected events were not reported during the clinical course of any of the patients in whom the r-Hb measurement was omitted.

Forty-six patients (5%) with a normal i-Hb and who did not fulfil the criteria for haemodynamic instability underwent an emergent surgical or angiographic procedure to control haemorrhage. The majority of these patients (*n* = 37, 80%) suffered blunt trauma. Median GCS on admission was 15 (IQR = 11 to 15) and median ISS was 25 (IQR = 10 to 28). In 21 of these 46 patients (46%), the r-Hb was normal. In 18 patients (39%), an abnormal r-Hb was found corresponding with injuries diagnosed on radiologic imaging. The median difference between i-Hb and r-Hb in these 18 patients was 1.21 g/dl (IQR = 0.77 to 1.53) and the median decrease in Hb concentration was 8.52% (IQR = 5.45 to 11.12). There were seven patients (15%) in whom no r-Hb with 2 h was performed, but unsuspected events were not reported.

## Discussion

This study demonstrates that repeated blood haemoglobin measurement within 2 h in adult trauma patients with a normal initial blood Hb concentration is normal in 70% of the patients. The repeated Hb measurement did not have value in the trauma work-up in our centre. In the remaining 30% of the patients, the repeated blood Hb concentration was abnormal or the measurement was not done at all, but in none of the patients, this had clinical consequences.

In today's medical practice, evidence-based medicine is an obvious and important basis for patient care. It is an interesting observation, that in our academic Level 1 trauma centre, the r-Hb measurement in most trauma patients is based on a historical habit without solid scientific evidence [11]. Furthermore, in 194 out of 1,243 patients (16%) admitted to our trauma room in a period of 2 years, the r-Hb was not done at all.

We hypothesized that the need for a r-Hb measurement was based on the hypothesis that a decrease in blood Hb concentration may indicate injury that is not otherwise detected. However, these Hb values should always be seen in the context of the clinical course and initiated therapy. Furthermore, in our centre, total-body CT scanning is performed on specific indications (pre-defined vital parameters, trauma mechanisms and injuries) [9]. It is well possible that with this imaging strategy, reasons for a drop in Hb becomes clearer than with FAST and X-rays alone. This is especially important in non-teaching hospitals, in which not even a FAST is obtained in every trauma patient. In these patients, the physician that is performing the trauma survey might find it reassuring to obtain a repeat Hb. However, our study shows that even when a repeat Hb is not obtained, or when it is abnormal in the absence of severe injuries, it is unlikely that there are clinical consequences. As a result of this study, in our centre, a repeat Hb level is only obtained at the discretion of the physician. Previous studies examined the relationship between laboratory results and mortality in trauma patients. Lam et al. [2] found significant differences in complete blood count (CBC) parameters between survivors and non-survivors and between single and multiple trauma patients in 1,673 patients. However, most of the CBC parameters demonstrated poor to moderate predictive ability for 7-day in-hospital mortality in adult trauma patients. Vroonhof et al. [12] demonstrated that nine laboratory parameters (ionized calcium, potassium, sodium, glucose, lactate, pH, pCO(2), pO(2) and saturation) were related to mortality in 1,806 trauma patients. Furthermore, haemorrhage in trauma patients was shown to be associated with a decrease in Hb levels with the first 30 min after patient arrival [13]. However, none of these studies reported on the value of repeated blood Hb measurements in relation to occult injuries or clinical decision making. We found that in none of the patients with a normal i-Hb, the r-Hb had clinical consequences, even in patients who underwent an emergent surgical or angiographic procedure to control haemorrhage.

### Limitations

A limitation of this study is that we retrospectively analysed prospectively collected data. We do not know to what extent crystalloid resuscitation received in the field influenced the value of the Hb measurement. Pre-hospital crystalloid resuscitation is generally not well documented and could therefore not be retrieved. This could explain the fact that in 81 patients, the cause of the decrease in Hb remained unclear and that no unsuspected events were reported despite the abnormal r-Hb. Furthermore, nothing is known about the motivation to omit the r-Hb measurement or which clinical consequences are correlated to a decrease in Hb. It would have been interesting to know whether a drop in Hb lead to placement of the patient in a monitored area, whether a drop in Hb resulted in a change in frequency of vital sign observation or at what time the decision made was made to take patients to either theatre or intervention in relation to time of second Hb measurement.

Another limitation of this study is the lack of information on the exact amount of packed red blood cells received by the patient during the first 2 h of admission. This is not routinely registered in the trauma registry and reliable data could not be retrieved retrospectively. In our centre, blood transfusion is given when there is a clinical suspicion for haemorrhage, the patient has a systolic blood pressure below 90 mmHg and is a non-responder to resuscitation fluids. A joint taskforce of the Eastern Association for Surgery of Trauma (EAST) developed a practice management guideline for red blood cell transfusion in adult trauma and critical care [14]. One of the recommendations is to consider blood transfusion if the Hb level is 7 g/dl or below in resuscitated critically ill trauma patients. In our cohort, however, none of the patients with an initial normal Hb had a repeated Hb below 9 g/dl. It is therefore less likely, but not impossible, that the amount of blood transfusions influenced the measured Hb levels.

It would be interesting to perform a prospective study on the value of repeated blood Hb measurements, also considering the selective population of trauma patients for which the repeated Hb might still be useful. This could lead to less r-Hb measurements which could decrease health care costs. In our centre, the cost of one Hb measurement is €6.65 (comparable to $8.28). Omitting the r-Hb in our study population (914 HD stable patients with a normal i-Hb) would thus have saved approximately €4,083 ($7,568) in a 2-year period.

## Conclusions

We conclude that repeated blood Hb measurement within 2 h after the initial Hb measurement in adult trauma patients with a normal initial blood Hb concentration and without evidence of haemodynamic instability does not add value to the current trauma work-up in our centre.

## Competing interests

The authors declare that they have no competing interests.

## Authors’ contributions

JCS, PJ and SMMdC carried out the analysis and drafted the manuscript. NWLS provided statistical expertise. JCG supervised the study. All authors were involved in the study design read and approved the final manuscript.
